# Physiological and Transcriptomic Responses to Nitrogen Deficiency in *Neolamarckia cadamba*

**DOI:** 10.3389/fpls.2021.747121

**Published:** 2021-11-23

**Authors:** Lu Lu, Yuanyuan Zhang, Lu Li, Na Yi, Yi Liu, Mirza Faisal Qaseem, Huiling Li, Ai-Min Wu

**Affiliations:** ^1^State Key Laboratory for Conservation and Utilization of Subtropical Agro-Bioresources, Guangdong Key Laboratory for Innovative Development and Utilization of Forest Plant Germplasm, College of Forestry and Landscape Architecture, South China Agricultural University, Guangzhou, China; ^2^Guangdong Key Laboratory for Innovative Development and Utilization of Forest Plant Germplasm, College of Forestry and Landscape Architectures, South China Agricultural University, Guangzhou, China; ^3^Guangdong Laboratory of Lingnan Modern Agriculture, Guangzhou, China

**Keywords:** nitrogen deficiency, *Neolamarckia cadamba*, transcriptome analysis, physiology, stress response

## Abstract

Nitrogen (N) is one of the abundant and essential elements for plant growth and development, and N deficiency (ND) affects plants at both physiological and transcriptomic levels. *Neolamarckia cadamba* is a fast-growing woody plant from the Rubiaceae family. However, the physiological and molecular impacts of ND on this species have not been well investigated. Here, we studied how *N*. *cadamba* responds to ND under hydroponic conditions. In a physiological aspect, ND led to a reduction in biomass, chlorophyll content, and photosynthetic capacity. ND also impaired the assimilation of N as the activities of glutamine synthetase (GS) and nitrate reductase (NR) were decreased in the root. Interestingly, the lignin content of stem increased progressively during the ND stress. The main transcription factors, the transcription factors that are important to N regulation has been found to be upregulated, including Nodule inception-like protein 7 (*NLP7*), TGACG motif-binding factor 1 (*TGA1*), basic helix-loop-helix protein 45 (*BHLH45*), NAM, ATAF1,2, CUC2 (NAC) transcription factor 43 (*NAC43*), and basic leucine zipper pattern 44 (*bZIP44*). The expression of N transporters, such as nitrate transporter 2.4 (*NRT2*.*4*), ammonium transporter 3 (*AMT3*), and amino acid transporter protein 3 (*AAP3*), was also upregulated. In addition, phosphorus- and calcium-related genes such as phosphate starvation response 2 (*PHR2*) and cyclic nucleotide-gated ion channel 15 (*CNGC15*) were expressed more abundantly in response to ND stress. Our results reveal the physiological and molecular mechanisms by which woody plants respond to ND.

## Introduction

Nitrogen (N) is the most important inorganic nutrient in plants. It is a major component of proteins, nucleic acids, cofactors, and secondary metabolites. N influences all aspects of plants, for example, metabolism, resource allocation, growth, and development (Stitt and Krapp, 1999). High N availability is required to ensure high productivity of crops, including woody crops such as fast-growing Poplars (Mamashita et al., 2015). However, in natural ecosystems and agroecosystems, the availability of N is often limited, leading to abiotic plant stress—nitrogen deficiency (ND) (Martin et al., 2002). Large amounts of N fertilizer are being used in agricultural soils to meet the increasing demand for food, raw materials, and biofuels from a growing global population (Robertson and Vitousek, 2009). About 75% of N fertilizer is not taken up by plants and is lost to the environment, leading to an eutrophication of water bodies and an enrichment of atmospheric N oxide gases (Gutierrez, 2012). To maintain high yields and reduce N application rates, it is necessary to better understand the molecular mechanisms that regulate the morphological and physiological adaptations of crops to N effectiveness (Rennenberg et al., 2010; Miller et al., 2012).

In nature, N in the soil exists mainly in two forms: organic and inorganic N. Urea, amino acids, and amides are examples of organic N. Plants, on the other hand, primarily absorb N in inorganic forms such as nitrate nitrogen (NO_3_^–^) and ammonium nitrogen (NH_4_^+^). N uptake by plants is mainly dependent on nitrate transporters (NRTs) and ammonium transporters (AMTs). Plant NRT proteins were discovered and functionally described more than two decades ago. They are encoded by at least four gene families, NRT1 (NPF), NRT2, CLC, and SLAC1/SLAH (Chopin et al., 2007; Geiger et al., 2009; Feng et al., 2011; Maierhofer et al., 2014; Qin et al., 2019). N assimilation is the process by which plants absorb inorganic N from the environment and then undergo a series of reactions within the plant to synthesize amino acids. The amount of NO_3_^––^ absorbed by plant cells is reduced to nitrite by cytoplasmic nitrate reductase (NR) (Masclaux-Daubresse et al., 2010) and delivered into the chloroplast, where it is converted to NH_4_^+^ by nitrite reductase (NiR) before being further utilized. Ammonium, either absorbed directly from the soil or converted from nitrate, is assimilated *via* a glutamine synthetase (GS)/glutamate synthase (GOGAT) cycle (Lam et al., 1996).

Nitrogen deficiency affects crop growth and development, with symptoms, including leaf loss, plant dwarfing, thickening of root cell walls, and reduced yield (Coruzzi and Bush, 2001; Papadopoulos and Pougioula, 2010; Kusano et al., 2011). Reduced chlorophyll content, decreased anthocyanin accumulation in leaves, and restricted root growth, particularly of lateral roots, were typical signs in young seedlings (Scheible et al., 2004). Recent studies in maize have shown that the gene regulatory network controlling the root development also has a role in ND response (He et al., 2016). In N-deficient plants, there was an increase in reactive oxygen species (ROS), which caused damages to the cell membranes. To cope up with ROS, plants actively produced ROS scavengers such as the superoxide dismutase (SOD) and the peroxidase (POD) (Yang et al., 2019). In addition, ascorbic acid synthesis was increased in cucumber seedlings and fruits under ND conditions, supporting the role of ROS in ND response (Zhao et al., 2015). N stress results in reduced amino acid synthesis, protein biosynthesis (Liu et al., 2019), and reduced activities of NR, GS, and GOGAT in the leaves and roots of potato (Zhang et al., 2020). The response of leaf photosynthesis to ND is related to chlorophyll. Because N is a raw material for chlorophyll and its biosynthetic enzymes, ND resulted in reduced chlorophyll production and, as a result, a decrease in leaf chlorophyll content (Mu et al., 2016). In plant leaves, photosystems use approximately half of N, while the light harvesting system, dominated by chlorophyll a and b, consumes the other third (Martin et al., 2007). The quantity of N in the light harvesting system of maize leaves was reduced under ND conditions. The photosynthetic rate was reduced in N-deficit plants, and the light energy absorbed by the light trapping antennae cannot be fully utilized for CO_2_ fixation, so the remaining excitation energy was lost through thermal dissipation, photorespiration, and cyclic electron transfer (Frank et al., 2001; Jiang et al., 2006). A transcriptome study of cucumber leaves subjected to ND was carried out to better understand the putative regulatory mechanisms of chlorophyll reduction and cell wall remodeling. ND suppressed the transcription of chlorophyll a/b-binding proteins (CAB) genes, hence increasing chlorophyll breakdown, whereas the BR signaling system regulated cucumber cell wall reformation (Zhao et al., 2015). In addition, previous findings have shown that hormone signaling is involved in the regulation of N metabolism (Ristova et al., 2016). In conclusion, there are a wide variety of changes in the different aspects of plants under ND stress.

The main transcription factors involved in the regulation of nitrate transport were *Nodule inception-like protein 7* (*NLP7*), *NLP6*, *TGACG motif-binding factor 1* (*TGA1*), *TGA4*, *Arabidopsis nitrate regulated 1* (*ANR1*), *basic leucine zipper 1* (*bZIP1*), *LOB domain-containing protein 37* (*LBD37*), *LBD38*, *PCF/TCP-domain family protein 20* (*TCP20*), *NAC domain-containing protein 4* (*NAC4*), *squamosa promoter binding protein-like 9* (*SPL9*), etc. (O’Brien et al., 2016). *APETALA2/ethylene responsive factor* (*AP2/ERF*), *WRKY*, basic helix-loop-helix protein (*BHLH*), *NAC*, *and MYB* transcription factors, which were associated with abiotic stress in plants and were also frequently expressed at elevated levels under ND conditions (Lea et al., 2007). A high proportion of ND tolerance-related transcription factors were found to be differently expressed in the maize and spinach as revealed by transcriptome analysis, particularly from APETALA2/ethylene responsive element binding proteins (AP2/EREBP), BHLH, and WRKY families. These transcription factors may play an important role in plant N stress (Chen et al., 2015; Joshi et al., 2020). Phosphorus and calcium response genes were also found to be involved in the nitrate response. NIN-LIKE PROTEIN 7 (NLP7) was a major regulator of the nitrate-dependent transcriptional response (Marchive et al., 2013; Alvarez et al., 2020) and nitrate assimilation process (Castaings et al., 2009; Konishi and Yanagisawa, 2013; Marchive et al., 2013). In Arabidopsis, CBL-interacting protein kinase 23 (CIPK23) and CALCINEURIN B-LIKE PROTEIN (CBL1/9) regulate NRT1.1/NPF6.3 phosphorylation to transport nitrate, which in turn promotes calcium influx into the cell. Calcium-dependent protein kinases (CPK10/30/32) promote NLP7 phosphorylation (Wang et al., 2021). At the low levels of nitrate, SPX (SPX domain-containing proteins) inhibits the expression of N- and phosphorus-responsive genes by binding to NLP3/phosphate starvation response 2 (PHR2) to block the entry of NLP3 and PHR2 into the nucleus. In rice, NRT1.1B, the E3 ligase NBIP1 and SPX4 form a complex at high levels of NO_3_^–^ conditions that leads to the ubiquitination and degradation of SPX4, thereby allowing NLP3 to enter the nucleus (Hu et al., 2019). The gene expression of Arabidopsis NLP7 and rice NLP3 induces nitrate response in the nucleus (Fichtner et al., 2021).

*Neolamarckia cadamba* is a tall evergreen tree that grows extremely fast (Liu et al., 2021), widely distributed in tropical and subtropical regions (such as South China, Indonesia, India, and Malaysia), intolerant of low temperatures and susceptible to insect damage. At the World Forestry Congress in 1972, it was recognized as a “miracle tree” for fast growth. Moreover, the tree provides an ideal raw material for plywood, fiberboard, pulp, and paper (Zhao et al., 2014, 2017; Ouyang et al., 2016). *Neolamarckia cadamba* has received more attention in recent years as a source of biomass for bioenergy production (Zhao et al., 2017).

However, as a fast-growing species, *N*. *cadamba* has a high demand for water and nutrients (Chu et al., 2017), and adequate nutrition is critical for its normal growth. So, in this study, the response of *N*. *cadamba* to ND stress was investigated at the physiological and molecular level. Specifically, differences in the transcriptome profiles of the roots and leaves of *N*. *cadamba* seedlings upon ND stress of 6 and 12 days were studied using RNA-sequencing (RNA-seq) to investigate the mechanism of fertilizer utilization. To our knowledge, this study is the first comprehensive report on the transcriptomic response of *N*. *cadamba* to ND stress.

## Materials and Methods

### Plant Materials and Nitrogen Deficiency Treatment

The experiments were conducted in the growth room at 25°C with 16 h light and 8 h dark. Some excellent clones of *N*. *cadamba* were obtained through a large-scale selection. Then, we picked up the best one for tissue culture. All seedlings used here were reproduced by tissue culture techniques for the propagation of those clones. The seedlings were cultured in a rooting medium for 15 days and then grown on outdoors for 7 days. The 4–5 cm tall seedlings were removed from the flasks, rinsed with tap water, and then fixed in a 6-L hydroponic box (length: 42 cm, width: 29 cm, and height: 7.5 cm) with a 1 cm^3^ volume sponge and foam board and placed in a growth room. The plants were incubated for 7 days in 1/4 Hoagland nutrient solution after 7 days of incubation in tap water as described by Dai et al. (2020) and Liu et al. (2021). The uniformed plants were finally selected and transferred into hydroponic boxes (length: 39 cm, width: 12 cm, and height: 10 cm) with a capacity of 2.5 L. Each box was fixed with a volume of 1 cm^3^ sponge and foam board to fix 5 seedlings, 5 seedlings for one replicate, and three replicates each for the control and treatment groups at the 6th and 12th day, so 60 seedlings in total once time were aerated by an oxygen pump (10 h/day) and changed every 3 days. The nutrient solution was changed every 3 days. The nutrient solution was composed of 4 mM Ca (NO_3_)_2_⋅4H_2_O, 6 mM KNO_3_, 2 mM MgSO_4_⋅7H_2_O, 1 mM NH_4_H_2_PO_4_, 80 μM NaFe-EDTA, 46.3 μM H_3_BO_3_,95 μM MnSO_4_⋅4H_2_O,0.8 μM ZnSO_4_⋅7H_2_O_2_, 0.3 μM CuSO_4_, and 0.02 μM (NH_4_)_6_Mo_7_O_24_⋅4H_2_O. For ND treatment, Ca (NO_3_)_2_⋅4H_2_O and KNO_3_ were replaced with CaCl_2_ and KCl for the bulk elements, NH_4_H_2_PO_4_ was replaced with KH_2_PO_4_, and (NH_4_)_6_Mo_7_O_24_⋅4H_2_O was removed, pH = 5.8. The concentration of N in the nutrient solution for the ND treatment was 0 mM, and the total nitrogen (CK) nutrient solution was 15 mM. Leaves and roots were taken at the 6th and 12th day for physiological index measurements and transcriptome sequencing. All experiments were repeated at least three times.

### Measurement of Plant Physiological Parameters

Samples were randomly taken from plants after 6 and 12 days of the normal and ND treatments, plant height was measured with a straightedge, data were recorded and photographs were taken to observe the phenotype; root architecture was scanned with a root scanner (Epson Expression 12000XL Scanner, Nagano, Japan) and root structure (root length, root volume, and root surface area) was analyzed with the Win RHIZO root analysis software.

The seedlings to be tested were rinsed three times with deionized water, blotted dry with paper towels, weighed as a whole, and then placed in kraft bags to be baked in an oven to a constant weight and weighed as a dry weight. Each indicator was measured in three replicates.

The enzyme activities of leaves and roots were determined using SOD, POD, and ascorbate oxidase (APX) content kits (Suzhou Comin Biotechnology Co., Suzhou, China) (Yao et al., 2017).

The first, second, and third stem nodes (counting from top to bottom) of 6- and 12-day seedlings were embedded in 3% (mass fraction) agarose, sliced to a thickness of 40 μm using a vibrating microtome (Leica VT1000S, Nussloch, Germany) and placed on slides, stained with 10% KOH for 4 min, rinsed three to five times with pure water, stained with 1M HCl for 4 min, and rinsed three to five times with pure water. The sections were then stained with 0.02% toluidine blue for 10 s and rinsed three times with pure water. The films were observed and photographed under an automatic digital scanning imaging system (Beijing Wanbang Junyi M8, Beijing, China).

### Chlorophyll Content and Fluorescence Determination

The chlorophyll fluorescence of leaves was measured *in vivo* using an IMAGING-PAM chlorophyll fluorescence imaging system (WALZ, Effeltrich, Germany) on three representative plants, avoiding the leaf veins as much as possible. The measurements were repeated three times, and the fluorescence parameters were analyzed.

The chlorophyll content of the leaves was determined by a 80% acetone extraction. About 0.1 g of the second pair of old leaves of the plant was taken and weighed in a 10-ml centrifuge tube after removing the veins, 5 ml of 80% acetone was added, and the leaves were soaked for 1 week under dark conditions until they turned white, and the chlorophyll content was determined by spectrophotometry (Wellburn, 1994). Three replicates of each index were determined.

### Indicators of N Metabolism Measurement

After dry weight measurement, 0.1 g of the tissue was weighed to determine the CK and protein content using Kjeldahl N determination (Hong et al., 2013). Fresh samples were obtained by random sampling of normal and N-deficient plants at the 6th and 12th day, then 0.1 g of the tissue was ground in liquid N and weighed to determine the activity of enzymes related to N metabolism using NR and GS kits (Suzhou Comin Biotechnology Co., Suzhou, China).

### RNA Extraction, Sequencing, and Differential Expression Analysis

Plants treated with ND and CK for 6 and 12 days were randomly sampled in three replicates per treatment to obtain leaf and root tissues, which were ground to a powder using liquid N. Then, the total RNA was extracted using the RNAprep Pure Assay kit (TIANGEN, Beijing, China). RNA quality was examined by electrophoresis using a 1% agarose gel and a NanoPhotometer^®^ spectrophotometer (IMPLEN, Westlake Village, CA, United States) and the Bioanalyzer 2100 system’s RNA Nano 6000 assay kit (Agilent Technologies, Santa Clara, CA, United States) was used to assess, respectively, the purity and integrity of the RNA. Transcriptome analysis was commissioned to Novogene Company (Beijing, China). The RNA-seq analysis was performed using the edgeR package in R software v. 3.18. Differential expression analysis for both treatments was performed by the edgeR package in R software v. 3.18. with the Benjamini and Hochberg method to adjust for significant values, using a differential expression threshold of the value of *p* < 0.05. TBtools was used to depict a heat map based on the differentially expressed gene (DEG) results (Chen et al., 2020). In addition, RNA-seq data were submitted to https://ngdc.cncb.ac.cn/gsa/browse/CRA004153 with the submission number: CRA004153.

### Functional Enrichment Analysis

Gene Ontology (GO) functional enrichment analysis of the DEG set was performed using the *ClusterProfile* software. The Kyoto Encyclopedia of Genes and Genomes (KEGG) pathway enrichment analysis was performed on the differential expressed gene sets using *ClusterProfile* package in R software, a comprehensive database that integrates genomic, chemical, and phylogenetic information. The threshold of significant enrichment was *p* < 0.05.

### Quantitative Real-Time-PCR Analysis

TaKaRa PrimeScript^TM^ RT reagent kit with gDNA Eraser (Perfect Real Time, Takara, Japan) was used to synthesize complementary DNA (cDNA) for real-time quantitative PCR (qRT-PCR) on a LightCycler 480 instrument (Beiqing et al., 2016). The information of primers for the genes for quantitative PCR (qPCR) are listed in Supplementary Table 3. SAMDC was chosen as the housekeeping gene (Dai et al., 2020). The expression data were calculated by the 2^–ΔΔCt^ method (Pfaffl, 2001).

## Results

### Morphology and Growth Parameters of *Neolamarckia cadamba* Upon Nitrogen Deficiency

**FIGURE 1:**
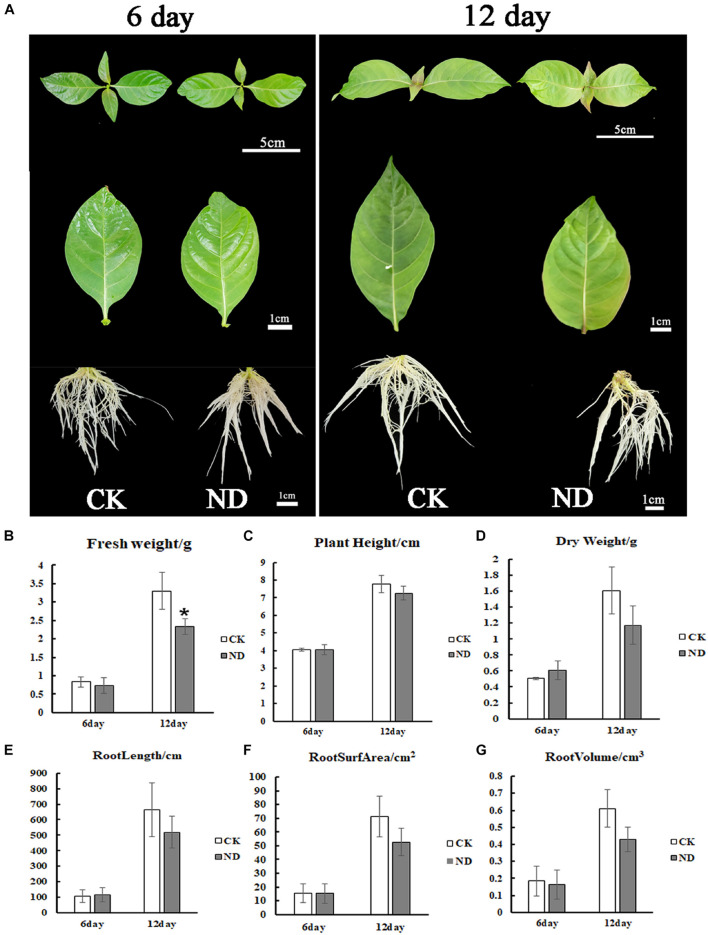
The growth of *Neolamarckia cadamba* under treatments of normal nutrition [total nitrogen (CK)] and nitrogen deficiency (ND). **(A)** The phenotypes of *N*. *cadamba* under total nitrogen (CK) and ND treatments in a growth room were shown at the 6th and 12th day, respectively. Fresh weight **(B)**, plant height **(C)**, dry weight **(D)**, root length **(E)**, root surface area **(F)**, root volume **(G)** under CK and ND conditions were determined at the 6th and 12th day, respectively. Compared with CK, although all the parameters were reduced under ND treatment at the 12th day, only fresh weight showed a significant decrease. Data are means ± SD (*n* = 3). Differences between the mean values of CK and ND were compared using the *t*-test (* denote significant differences at *p* < 0.05).

**FIGURE 2:**
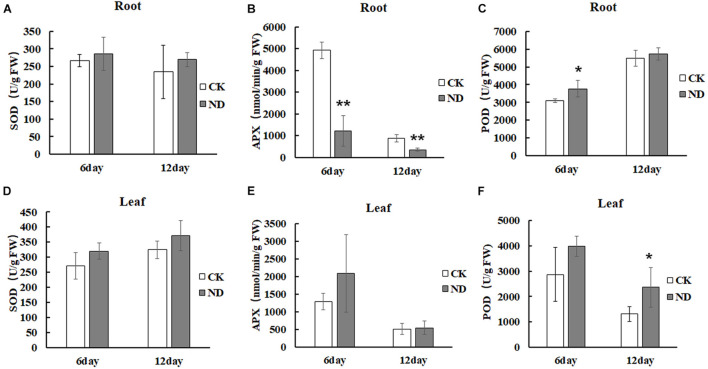
Enzyme activity in roots and leaves, superoxide dismutase (SOD) **(A,D)**, ascorbate oxidase (APX) **(B,E)**, peroxidase (POD) **(C,F)** was determined at indicated times. Enzymatic activities were measured in roots and leaves under the ND and CK treatment. Compared with CK, there was a significant difference in the physiological parameters of ND at the last stages. Data are means ± SD (*n* = 3). Differences between the mean values of CK and ND were compared using *t*-test (* and ** denote significant differences at *p* < 0.05 and *p* < 0.01).

To evaluate the effects of ND stress on the plant growth, *N*. *cadamba* seedlings were investigated. At the 6th day, the root length under ND stress was the same as in the CK but with fewer lateral roots, while at the 12th day, the root length was shorter than in the control and leaves started to fade from fresh green to yellow (Figure 1A). The biomass of *N*. *cadamba* seedlings was also examined (Figures 1B–G). In the ND treatment group, fresh weight, dry weight, plant height, and root system (length, surface area, and volume) were not significantly different from the control at the 6th day. All these physiological indicators were lower than those in the control (CK) at the 12th day, with fresh weight reduced by 11.3%, dry weight reduced by 27%, plant height reduced by 6.5%, and root length, root surface area, and root volume reduced by 21.9, 25.9, and 29.6%, respectively (Figures 1B–G). The antioxidant enzyme activity in *N*. *cadamba* also responded differently to ND stress (Figures 2A–F). SOD and POD activities were affected differently depending on tissues and the time duration under ND stress. To be specific, POD was most significantly affected in roots at the 6th day and leaves at the 12th day as its content increased by 21.1 and 79.8%, respectively, while APX, on the other hand, decreased significantly in roots, by 75.2 and 59.2% at the 6th and 12th day, respectively (Figures 2A–F).

### Transcriptome Changes in Response to Nitrogen Deficiency

**FIGURE 3:**
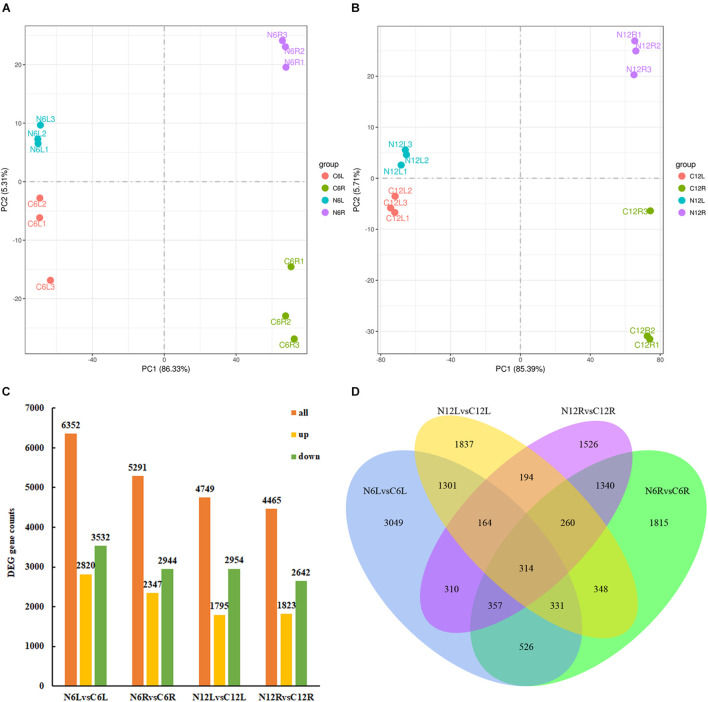
Differentially expressed genes (DEGs) in *N*. *cadamba* of control and ND conditions. The transcriptome of *N*. *cadamba* were sequenced and analyzed from the different samples of roots and leaves under ND and CK. Then, principal component analysis (PCA) and DEGs were analyzed. **(A)** PCA of DEGs at the 6th day. **(B)** PCA of DEGs at the 12th day. **(C)** The orange bar represents all DEGs, downregulated DEGs are in green, and upregulated DEGs are in yellow, FDR < 0.05. **(D)** This Venn diagram converges the DEGs of CK vs. ND at different time points (6 and 12 days) and different tissues (root and leaf). The overlapping area represents the DEGs with a common regulation mode between different treatments.

Total RNA was extracted from the roots and leaves of *N*. *cadamba* at the 6th and 12th day, and eight data sets were obtained, namely N6L, C6L, N6R, C6R, N12L, C12L, N12R, and C12R for transcriptome sequencing. The low quality and short reads were removed. Clean reads mapped over 75% of the *N*. *cadamba* reference genome (Supplementary Table 1). Principal component analysis (PCA) clustered transcripts into four groups according to tissues and treatments, indicating that there were good correlations and differences among the samples (Figures 3A,B).

The comparative transcriptome analysis of *N*. *cadamba* under ND stress provided a basis for further elucidation of gene function and metabolic pathways. The number of DEGs (*p* ≤ 0.05) between groups differed significantly, i.e., 6,352 DEGs for N6L vs. C6L, 5,291 for N6R vs. C6R, 4,749 for N12L vs. C12L, and 4,465 for N12R vs. C12R (Figure 3C). The significant DEGs at different times and tissues under ND stress were used in the functional enrichment analysis, i.e., GO and KEGG databases. The corrected *p* ≤ 0.05 was defined as prominent enriched GO terms (Supplementary Figure 1) response to oxidative stress (GO:0006979) and response to stress (GO:0006950) were significant GO terms associated with biological processes in roots, while in leaves GO terms associated with photosynthesis (GO:0015979), peptide biosynthetic process (GO:0043043), and amide biosynthetic process (GO:0043604) were significantly enriched. For cellular components, photosynthetic membrane (GO:0034357), photosystem (GO:0009521), and cell wall (GO:0005618) were enriched in roots while in leaves GO terms associated with ribosome (GO:0005840), thylakoid (GO:0009579), photosynthetic membrane (GO:0034357), and photosystem (GO:0009521) were significantly enriched. For molecular functions, heme binding (GO:0020037), peroxide activity (GO:0004601), and tetrapyrrole binding (GO:0046906) were significant GO terms in roots while structural molecule activity (GO:0005198), iron–sulfur cluster binding (GO:0051536), and metal cluster binding (GO:0051540) were significantly enriched in leaves.

After the KEGG enrichment analysis for CK and ND at the 6th day was completed, significant KEGG pathways enriched in roots were identified, including mainly phenylpropanoid biosynthesis, carbon fixation in photosynthetic organisms, N metabolism, MAPK signaling pathway-plant, and plant hormone signal transduction. While for leaf samples carbon fixation in photosynthetic organisms was the most significantly enriched pathway. Interestingly, in the 12 day samples, porphyrin and chlorophyll metabolism, carbon fixation in photosynthetic organisms, N metabolism, photosynthesis, and photosynthesis-antenna proteins were significantly enriched pathways in leaves while in roots phenylpropanoid biosynthesis, N metabolism, and MAPK signaling pathway-plant were significantly enriched.

There were 314 differential expressed genes that were shared by all ways of comparison (Figure 3D). We were mainly interested in DEGs associated with “carbon metabolism” and “N metabolism” pathways by KEGG, and GO terms of these DEGs were in oxidoreductase activity (GO:0016616) and hexose metabolic process (GO:0019318).

**FIGURE 4:**
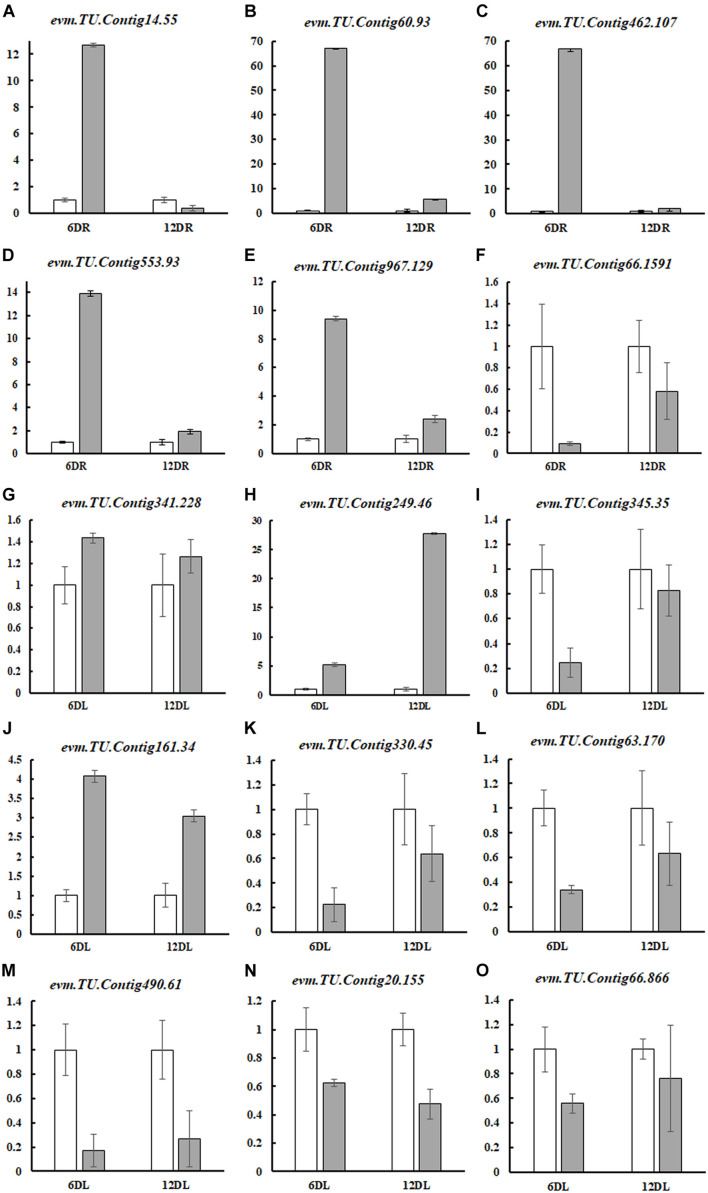
Quantitative real-time PCR validation of 15 DEGs. Normal nutrition control (CK), ND, root (R), and leaf (L) were labeled, and the pairs of CK and ND were compared at different time points (6DR: CKR6d vs. NDR6d; 12DR: CKR12d vs. NDR12d; 6DL: CKL6d vs. NDL6d; and 12DL: CKL12d vs. NDL12d). The experiments were repeated four times. The expression level of each gene in the CK was “1”. These genes included nitrate transporter (NRT) proteins (*evm*.*TU*.*Contig14*.*55*, *evm*.*TU*.*Contig60*.*93*, *evm*.*TU*.*Contig462*.*107*, *evm*.*TU*.*Contig66*.*1591*), hormone response (*evm*.*TU*.*Contig553*.*93*, *evm*.*TU*.*Contig967*.*129*), transcription factors (*evm*.*TU*.*Contig341*.*228*, *evm*.*TU*.*Contig249*.*46*), photosynthesis (*evm*.*TU*.*Contig345*.*35*, *evm*.*TU*.*Contig20*.*155*, evm.TU.Contig66.866), chlorophyll synthesis (*evm*.*TU*.*Contig330*.*45*, *evm*.*TU*.*Contig63*.*170*, *evm*.*TU*.*Contig490*.*61*), and amino acid transporter proteins (*AAPs*) (*evm*.*TU*.*Contig161*.*34*). Gene-specific primers used for real-time PCR are listed in Supplementary Table 3.

To confirm the transcriptome results, 15 genes were randomly selected for qRT-PCR analysis. These genes are responsible for nitrate transporting, hormone response, photosynthesis, chlorophyll synthesis, and amino acid transporter proteins (*AAPs*). qRT-PCR results demonstrated that all gene expression trends were consistent with our RNA-seq data, indicating that our transcriptome analysis was reliable and ready for further analysis (Figure 4).

### Chlorophyll Content and Photosynthetic Parameters Decreased Under Nitrogen Deficiency Stress in *Neolamarckia cadamba*

**FIGURE 5:**
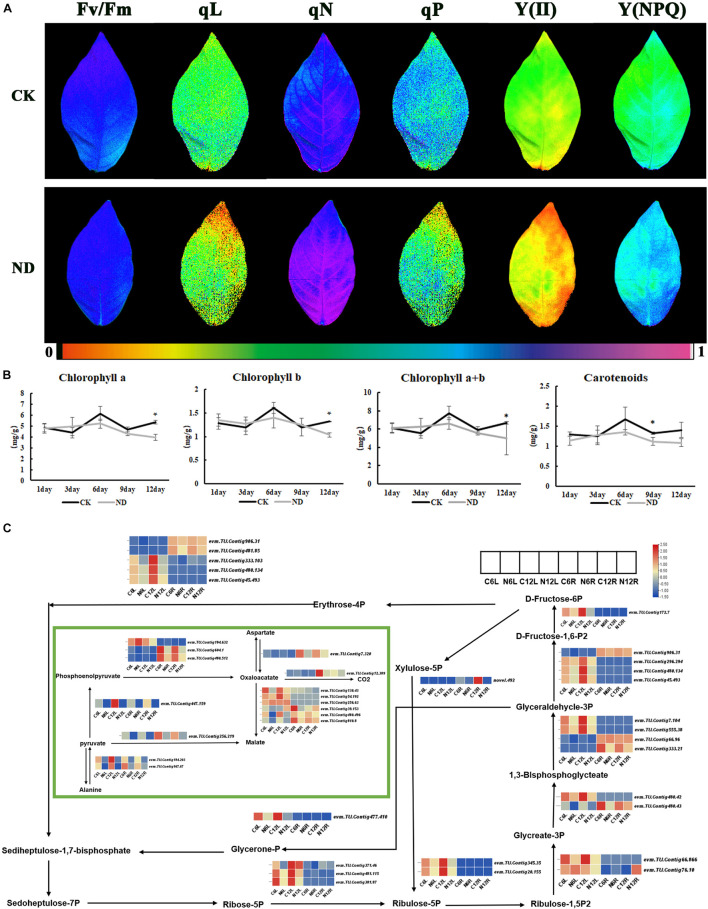
Response of ND to photosynthesis in *N*. *cadamba* at the physiological and molecular level. **(A)** The chlorophyll fluorescence imaging is shown in Fv/Fm, qL, qN, qP, Y(II), and Y(NPQ), which were examined at the 12th day. **(B)** The content of Chlorophyll a, Chlorophyll b, Chlorophyll a + b, and Carotenoids at 1, 3, 6, 9, and 12 days was measured. Data are means ± SD (*n* = 3). Differences between the mean values of CK and ND were compared using student’s *t*-test (* denote significant differences at *p* < 0.05). **(C)** RNA-sequencing (RNA-seq) data showed the DEGs of carbon fixation in photosynthesis pathway (KO00710). Heatmap of columns and rows represent samples and genes, respectively. Chlorophyll fluorescence parameters were listed in Supplementary Table 2.

As yellowing leaf was found in the treatment at the 12th day, the efficiency of leaf photosynthesis was measured by imaging-PAM (Figure 5A) and some important parameters related to photosynthesis, such as Y(I), ETR(I), Y(II), qN, qP, and qL, were also measured to evaluate the effects of ND on photosynthesis (Supplementary Table 2). It was obvious that Y(I) and ETR(I) were significantly reduced at both the 6th and 12th day under ND. At the 6th day, although the leaves did not turn yellow significantly as much as the 12th day, Y(II), qN, qP, and qL all decreased significantly (Supplementary Table 2). At the 12th day, besides Y(II), qN, qP, and qL, chlorophyll fluorescence imaging was also decreased. However, Y(NPQ) increased at the 6th and 12th day (Figure 5A). In addition, the contents of chlorophyll a, chlorophyll b, total chlorophyll, and carotenoids were measured at the 1st, 3rd, 6th, 9th, and 12th day, and the results showed that these contents under ND began to decrease at the 6th day, and the decrease reached a larger level at the 12th day after the yellowing of the leaves appeared (Figure 5B).

In the transcriptome DEGs data, the carbon fixation in photosynthetic organisms under ND stress was enriched with a high expression in leaves such as *evm*.*TU*.*Contig 333*.*103*, *evm*.*TU*.*Contig 480*.*134*, *evm*.*TU*.*Contig 45*.*493*, and *evm*.*TU*.*Contig 477*.*410*. At the same time, there were a few genes that were preferentially expressed in roots, such as *evm*.*TU*.*Contig 604*.*1*, *evm*.*TU*.*Contig 480*.*512*, and *evm*.*TU*.*Contig 256*.*219*. As expected, all these gene expressions were downregulated in ND comparing with CK (Figure 5C), suggesting that ND does affect photosynthesis.

### Nitrate Reductase, Nitrite Reductase, Glutamine Synthetase, Nitrogen Content, and Nitrogen Metabolism Response to Nitrogen Deficiency Stress

**FIGURE 6:**
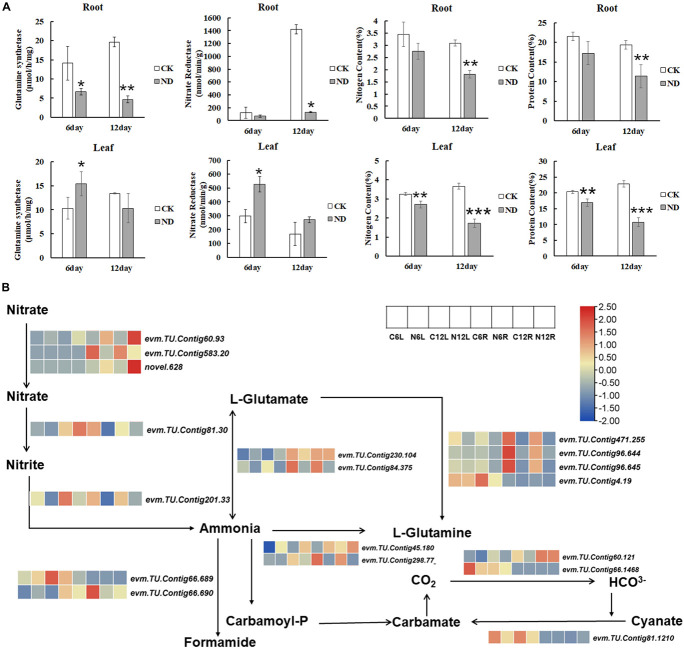
Nitrogen (N) metabolism in response to ND in *N*. *cadamba*. **(A)** The changes of the *N*. *cadamba* under CK and ND treatments in glutamine synthetase (GS), nitrate reductase (NR), N content, and protein content. Data are means ± SD (*n* = 3). Differences between the mean values of CK and ND were compared using student’s *t*-test (*, **, *** denote significant differences at *p* < 0.05, *p* < 0.01, and *p* < 0.001). **(B)** Transcriptome data showing the expression of genes related to N metabolism in *N*. *cadamba* after ND. A heatmap of columns and rows represent samples and genes, respectively.

To determine the responses of plants to ND, some important parameters participating in N metabolism, such as GS, NR, NiR, N content, and protein content, were measured (Figure 6A). NR and NiR catalyzed the reduction of nitrate to nitrite and nitrite to ammonia, respectively. So, NR is the first rate-limiting enzyme in the assimilation of nitrate into plants. Under ND stress, the activity of the NR kept falling in roots till 12 days while in leaves it significantly increased at the 6 day, implying that NR activities might have been compensated in leaves (Figure 6A). Further, *evm*.*TU*.*Contig 201*.*33*, which encodes NiR and was significantly downregulated under ND conditions at the 6th and 12th day (Figure 6B). GS and GOGAT can convert glutamate and ammonia to glutamine. In our measurement, the activity of the GS was significantly reduced at the 6th and 12th day in roots while in leaves it increased initially at the 6th day and then decreased at the 12th day (Figure 6B). The transcriptome analysis showed that the expressions of *evm*.*TU*.*Contig 45*.*180* and *evm*.*TU*.*Contig 298*.*77*, both encoding GS, were downregulated and consistent with the reduced GS activity phenotype. Meanwhile, *evm*.*TU*.*Contig 471*.*255*, *evm*.*TU*.*Contig 96*.*644*, *and evm*.*TU*.*Contig 96*.*645*, encoding GOGAT, were significantly downregulated in roots at both the 6th and 12th day, and *evm*.*TU*.*Contig 4*.*19* was significantly downregulated in leaves at the 12th day (Figure 6B).

As ND stress inevitably affects both the N and protein content of plants, these two components are measured. Compared to CK, the samples under ND showed a reduction of 16.61% in leaves and 19.94% in roots in the N content at the 6th day. At the 12th day, a reduction of 41.28% in roots and a reduction of 53% in leaves were observed. Because protein content was converted from N content, the changes of these two indicators were consistent.

### Relative Transporters and Transcription Factors Were Mainly Upregulated

**FIGURE 7:**
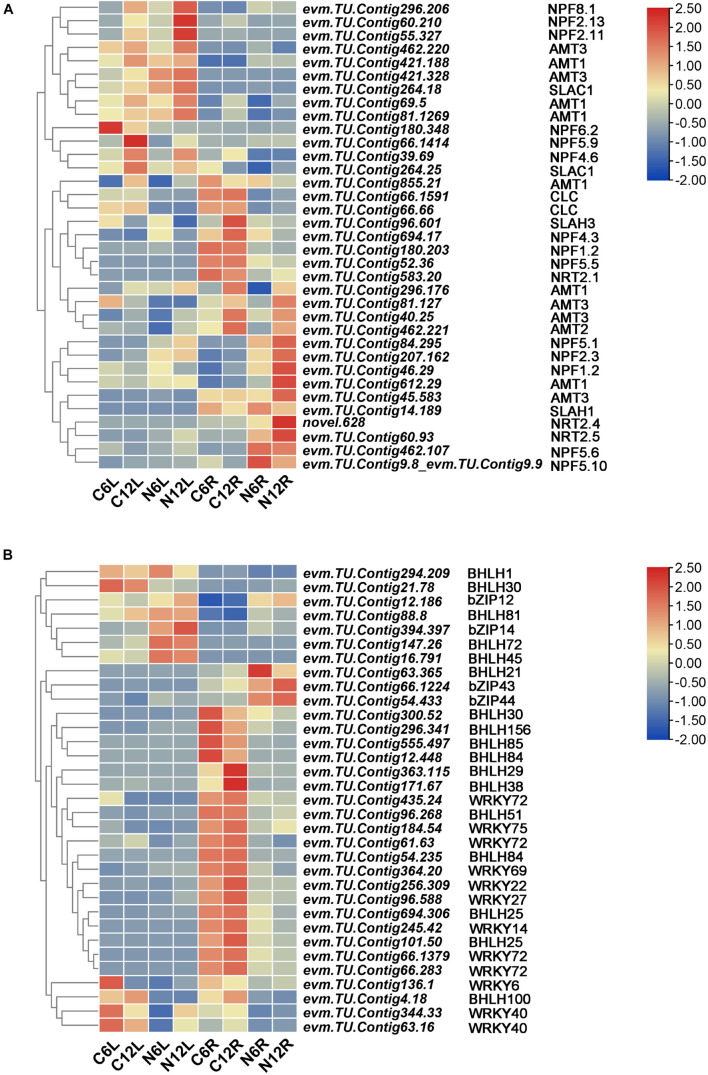
Nitrogen deficiency stress induces the expression of related transporter proteins and transcription factors in the leaf and root. **(A)** A heatmap of ND stress-induced expression of related transcription factors. **(B)** A Heatmap of NRTs and ammonium transporters (AMT) expression induced by ND stress. The threshold of FDR < 0.05, | log2 (FoldChange)| > 1 was used to screen the changes of DEGs between CK and ND in treatments. A heatmap of columns and rows represent samples and genes, respectively. Sample and DEGs names were displayed below the heatmap. The color bar is the scale for the expression levels of each gene.

Transporters are important factors related to N absorption. The DEGs identified showed that the NRTs, including *NRT1* (*NPF*), *NRT2*, *CLC* and *SLAC1*/*SLAH*, and AMT were upregulated in both roots and leaves under ND stress (Figure 7A). For example, *evm*.*TU*.*Contig 60*.*93*, *evm*.*TU*.*Contig 583*.*20*, and *novel*.*628*, all encoding *NRT2* genes, were upregulated under ND conditions (Figure 6B). At the same time, we also identified a number of amino acid transporters, such as *AAP7*, *AAP6*, *AAP3*, *ANTL1*, and *ANTL2*, which were mostly upregulated (Supplementary Figure 3A). *GABA* transporters, such as *GAT1* and *GAT2*, were also upregulated in response to ND stress in *N*. *cadamba*. In addition, auxin transporter-like protein (*LAX*), transmembrane *AAP*, vacuolar amino acid transporter 1, lysine histidine transporter (*LHT*), and vesicular inhibitory amino acid transporter (*VIAAT*) were upregulated.

To investigate changes in transcription factors upon ND stress, DEGs of transcription factors (*BHLH*, *bZIP*, *MYB*, and *WRKY*, etc.) at different tissues and time points under ND stress were identified. *BHLHs*, *bZIPs*, and *WRKYs* with 2-fold differences under ND were selected (Figure 7B). *BHLH1*, *BHLH30*, *bZIP12*, *bZIP14*, *bZIP43*, *bZIP44*, *BHLH81*, *BHLH72*, *BHLH45*, and *BHLH21* were upregulated, while the members of WRKY family were mostly downregulated. Additionally, transcription factors, including *NLP7*, *NLP6*, *TCP20*, *TGA1*, *ERF*/*RAP2*, and *ERF72*, were also responded to ND stress (Supplementary Figure 3B).

### Nitrogen Deficiency Stress Stimulates Hormone Gene Expression

Several plant hormones, such as salicylic acid (SA) and abscisic acid (ABA), have been reported to be associated with the metabolism and accumulation of N (Vega et al., 2019). In this study, DEGs (Supplementary Table 4) associated with hormones were identified, including nine auxin-related genes and five of which belong to the *PIN* family. *evm*.*TU*.*Contig 600*.*155* (*PIN2*) and *evm*.*TU*.*Contig 16*.*409* (*PIN2*) were downregulated in the root. *Novel*.*433* (*PIN5*) and *novel*.*551* (*PIN5*) were both upregulated in roots. In addition, genes related to gibberellin, cytokinin, ABA, and SA were also differentially expressed (Supplementary Table 4). This suggests that these hormones were engaged in the response of *N*. *cadamba* plants to ND.

### The Phenylpropanoid Biosynthetic Pathway Changes in Nitrogen Deficiency Stress

Stem sections at the 6th and 12th day revealed (Supplementary Figure 2A) an increase in lignification and cell wall thickening from the top to bottom of stem. In our transcriptomic data, KEGG pathway revealed an enrichment in “Phenylpropanoid biosynthesis” in roots. Further analysis of the genes in this pathway exhibited that *evm*.*TU*.*Contig 14*.*317* [4-coumarate-CoA ligase (*4CL*)], *evm*.*TU*.*Contig 296*.*60* [cinnamate 4-hydroxylase (*C4H*)], *evm*.*TU*.*Contig 14*.*271* [cinnamyl alcohol dehydrogenase (*CAD*)], *novel*.*2274* [*cinnamoyl-CoA reductase* (*CCR*)], *evm*.*TU*.*Contig 149*.*105* [*caffeic acid O-methyltransferase* (*COMT*)], *evm*.*TU*.*Contig 45*.*482* [ferulate 5-hydroxylase (F5H)], *evm*.*TU*.*Contig 766*.*86* [*phenylalanine ammonia-lyase* (*PAL*)], and POD protein genes were upregulated (Supplementary Figure 2B).

### The Response of Phosphorus- and Calcium-Related Genes to Nitrogen Deficiency in *Neolamarckia cadamba*

Nitrogen and phosphorus are the two mineral nutrients that are highly in demand by plants. N/P supply ratios affect N/P uptake by activating the phosphorus signaling pathway (Hu et al., 2019). The SPX domain-containing protein (*SPX*) gene, which regulates the cellular response to phosphate starvation, was significantly downregulated in leaves, but in roots, more *SPX* genes were upregulated more than downregulated (Supplementary Table 5). The majority of the phosphate transporter protein-related genes were downregulated.

Furthermore, Ca^2+^, a ubiquitous second messenger in plants, also plays a role in the nitrate response (Riveras et al., 2015). There were another expression trends in calcium-related genes. cyclic nucleotide-gated ion channel (*CNGC*) a cyclic nucleotide-gated channel with calcium channel activity, was downregulated in roots, just like the CPK and CBL-interacting protein kinase, while the CBL-interacting serine-/threonine-protein kinase was upregulated in leaves and roots.

## Discussion

Nitrogen is one of the most abundant elements and is required for the biosynthesis of amino acids, proteins, and enzymes in plant cells (Ganie et al., 2017). *Neolamarckia cadamba* is a fast-growing species, and its rapid growth must be dependent on an adequate supply of N. Therefore, ND certainly affects the rapid growth of *N*. *cadamba*, but previous studies have not elucidated the underlying mechanisms. Therefore, we combined the relative physiological parameters and transcriptome analysis to gain insights into the growth of *N*. *cadamba* on ND stress. The present study showed that the growth of *N*. *cadamba* seedlings was limited under ND. A reduction in fresh weight, dry weight, plant height, and root system (surface area, length, and volume) was recorded at the 12th day of ND treatment compared to CK (Figure 1), which was consistent with the *Eucalyptus* plants grown under low N conditions (Nguyen et al., 2003), indicating that N plays a critical important role in woody plant growth.

Photosynthetic capacity is an important indicator of plant growth (Blankenship, 2002; Zhang et al., 2014). In our study, although the maximal quantum yield of PSII photochemistry (Fv/Fm) did not change significantly between the ND and CK conditions, the photochemical quenching (qP) of the leaves of *N*. *cadamba* seedlings under ND stress was significantly lower than those of CK, and the actual photochemical efficiency of leaf PSII [Y(II)] and qL decreased vastly in ND, implying that the photosynthetic efficiency of *N*. *cadamba* leaves decreased in ND. Transcriptome analysis showed that the KEGG enrichment pathway for “porphyrin and chlorophyll metabolism” was downregulated in leaves after 6 days of ND stress, which is consistent with the downregulation of chlorophyll synthesis and carbon fixation in the chloroplast after 6 days of ND stress (Figure 5C). Although photosynthetic pathway under ND decreased at the 6th day, but the phenotype of yellowing leaf was not observed till the 12th day, suggesting a lag between the transcriptional changes and the phenotypic transition (Figure 1A). Meanwhile, the photosynthetic genes, such as *evm*.*TU*.*Contig 45*.*493* [fructose-bisphosphate aldolase 2 (*FBA2*)], *evm*.*TU*.*Contig 345*.*35* (*PRK*), *evm*.*TU*.*Contig 172*.*7* (*FBPase*), and *evm*.*TU*.*Contig 604*.*1* (*PEPC*), were downregulated in leaves under ND (Figure 5C), indicating that downregulation of photosynthesis-related genes under ND causes physiological yellowing of leaves. Furthermore, the reduction of dry weight in ND treatment could be related to photosynthesis inhibition, since less photosynthetic products were accumulated.

Transporters play critical roles in the first steps of N assimilation in plants. According to the transcriptome analysis, both NRT and AMT were upregulated to maintain N balance against external ND stress, which may indicate that the plant requires a higher expression of the N transporters to promote nitrate and ammonium accumulation. The activities of critical enzymes of N metabolism, such as NR, NiR, GS, and GOGAT, indirectly reflect the rate of the N assimilation and accumulation in plants (Zhang and Chu, 2020). *Evm*.*TU*.*Contig 81*.*30* (*NR2*), which can convert the nitrate to nitrite, was downregulated in roots compared to the CK (Figure 6B), consistent with the NR activity reduction in roots. The expression of N metabolism-related genes was downregulated in plants, implying that the scale of N assimilation was reduced, ultimately leading to a decrease in N and protein content in plants (Figure 6A). Under N-limiting conditions, Poplar induces transcripts of many N-transporter genes in their roots, allowing them to adapt to soil ND by reducing N assimilation (Luo et al., 2015; Meng et al., 2018). This also explained why the root biomass did not change significantly at the 6th day but started to decline at the 12th day. Furthermore, amino acid transporters, including *AAP3*, *GAT1*, and *LAX5*, were upregulated in response to ND stress in *N*. *cadamba* (Supplementary Figure 3A). Amino acid transporters’ response to ND has been previously identified in tea plants. The gene expression of these transporters increased under ND conditions suggesting that they occur in various organs (Li et al., 2020). The effects of ND on enzyme activity and amino acid transporters described above will ultimately reduce the N and protein content of *N*. *cadamba* (Figure 6A). Likewise, the reduction in N content is consistent with *Eucalypts* and Poplar at low N (Nguyen et al., 2003; Song et al., 2019).

Hormonal signaling has also been linked to N metabolism (Ristova et al., 2016). Here, the KEGG pathway “plant hormone signal transduction” was upregulated at the 6th and 12th day in the leaves of *N*. *cadamba* after ND stress, indicating that the hormone compensated to ND stress. We identified genes with | log_2_foldchange| > 1 (Supplementary Table 4) and found five hormones that responded to ND stress. The expression of SA-related gene (*SABP2*) was downregulated. SA was reported to reduce the nitrate accumulation caused by N starvation (Yendrek et al., 2010; Conesa et al., 2020). ABA and auxin are thought to be signaling molecules that bind to NO_3_^–^ and regulate root growth and development (Signora et al., 2001). *ABAH* and *PYL*, which are associated with ABA, were upregulated. *ARF* and *PIN5* both related to auxin were upregulated. In addition, *G2OX2* and *CKX3* were downregulated, suggesting that the gibberellin and cytokinin signaling pathways were also affected by ND stress (Supplementary Table 4).

Thinner fibrous walls were observed in the secondary xylem of hybrid poplars (*Populus trichocarpa* × *deltoides H11-11*) under high N conditions relative to plants with sufficient N (Plavcova et al., 2013). Our results showed an increase of lignification and thickening of the cell walls in the stem (Supplementary Figure 2). Root transcriptome data also revealed that most genes in the phenylpropanoid pathway were upregulated. Enzymes involved in phenylpropanoid metabolism, such as *4-coumaric acid-CoA ligase* (*4CL*), *CCR*, *COMT*, and *CAD*, were increased under N-limited conditions (Scheible et al., 2004). ROS can stimulate lignification and induce SOD and POD activities. In our experiments, ND treatment increased both SOD and POD activities in roots and leaves (Figure 2), which is consistent with the fact that ND stress promotes a significant increase in SOD and POD activities in cucumber leaves (Yang et al., 2019). Therefore, ND stress might stimulate lignification in plants but an exact mechanism is still not clear.

A previous study reported the main transcription factors that participate in the regulation of nitrate transport were Nodule inception-like protein 7 (*NLP7*), *NLP6*, *TGA1*, *TGA4*, *ANR1*, and *bZIP1*, etc. (O’Brien et al., 2016). Under N starvation, the expression of *BHLH*, a transcription factor, increases and mediates anthocyanin accumulation (Lea et al., 2007). In our study, the expression of transcription factors, such as *BHLH*, *bZIP*, *WRKY*, and *NLP7*, were induced (Figure 7B), while *WRKY40*, *WRKY72*, *WRKY22*, *WRKY27*, and WRKY14 were downregulated. Meanwhile, *NLP7*, *NLP6*, and *TGA1* were upregulated under ND condition in *N*. *cadamba* (Supplementary Figure 3B).

We discovered phosphorus- and calcium-related genes in our data (Supplementary Table 5), the *SPX* genes that were upregulated are more than those downregulated in roots at the 12th day, indicating that NLP3 cannot responsive to nitrate gene expression. Furthermore, *PHR2* was found to be upregulated, implying that phosphate-related genes work to alleviate the nutritional suppression caused by ND. Furthermore, *CNGC* and *CPK* were downregulated in roots. Under ND, CNGC15 and NRT1.1, which form a complex on the cytoplasmic membrane, lacked a calcium channel activity and were unable to encode a calcium channel for the nitrate nutrient response (Wang et al., 2021). It was proposed that ND caused dwarfism in *N*. *cadamba* and hampered its rapid growth, most likely due to the combined effect of Ca- and P-related genes (Supplementary Table 5).

**FIGURE 8:**
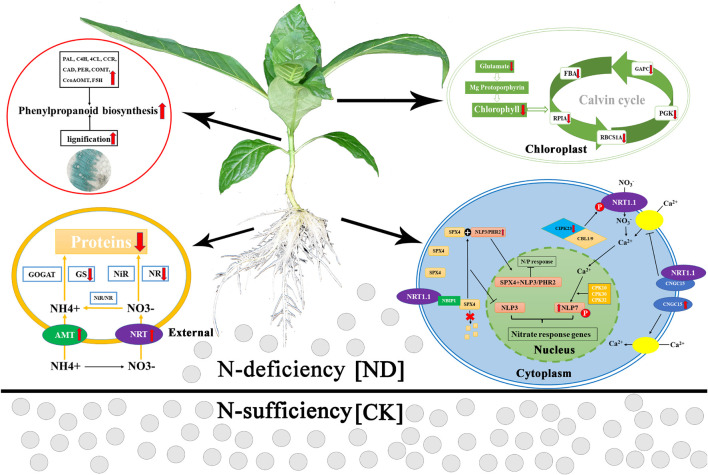
A hypothetical model of the effects of ND on the physiological and molecular mechanisms of *N*. *cadamba*. Phenotypes and transcriptome changes in roots, stems, and leaves treated with ND are indicated by the red arrows. Red arrows indicate an increase or a decrease in physiological indicators or gene expression. PAL, phenylalanine ammonia-lyase; C4H, cinnamate 4-hydroxylase; 4CL, 4-coumarate-CoA ligase; CCR, cinnamoyl-CoA reductase; CAD, cinnamyl alcohol dehydrogenase; COMT, caffeic acid *O*-methyltransferase; CCoAOMT, caffeoyl-CoA *O*-methyltransferase; F5H, ferulate 5-hydroxylase; NR, nitrate reductase; NiR, nitrite reductase; GS, glutamine synthetase; GOGAT, glutamate synthase; AMT, ammonium transporter; NRT, nitrate transporter; FBA, fructose-bisphosphate aldolase; GAPC, glyceraldehyde-3-phosphate dehydrogenase; PGK, phosphoglycerate kinase; RBCS1A, ribulose bisphosphate carboxylase small chain; RPIA, ribose 5-phosphate isomerase A; SPX4, SPX domain-containing proteins 4; NLP7/3, *nodule inception-like protein 7/3*; PHR2, phosphate starvation response 2; CBL1/9, calcineurin B-like protein; CIPK23, CBL-interacting protein kinase 23; CNGC15, cyclic nucleotide-gated ion channel 15.

Based on our results, we hypothesize the following pattern of the response to ND stress in *N*. *cadamba* (Figure 8). To compensate for ND stress, roots take up NH_4_^+^ *via* AMT and NO_3_^–^ *via* NRT from the soil, whose expression was upregulated. However, due to limited substrate availability, i.e., NH_4_^+^ and NO_3_^–^, the expression of the internal genes encoding the N assimilating enzymes (NR and GS) was decreased, resulting in a decrease in amino acid and protein biosynthesis. Meanwhile, under low N conditions, SPX4 forms a complex with NLP3/PHR2, preventing NLP3 and PHR2 from entering the nucleus and thereby inhibiting the expression of N and phosphorus-responsive genes in *N*. *cadamba*. The expression of *CNGC15* was upregulated to promote Ca^2+^ uptake. Therefore, these results suggested that N assimilation was linked with calcium and phosphorus absorption in *N*. *cadamba*. To deal with N stress, *N*. *cadamba* upregulated the expression of genes related to the phenylpropanoid biosynthetic pathway (including *PAL*, *C4H*, *4CL*, and *CCR*) in the stem and eventually increased its lignification. Decreased expression of glutamate and chlorophyll biosynthesis genes results in consecutive downregulation of the genes involved in the Calvin cycle, such as *FBA*, phosphoglycerate kinase (*PGK*), and ribulose bisphosphate carboxylase small chain (*RBCS1A*), resulting in the inhibition of leaf photosynthesis and thus in a decrease of *N*. *cadamba* fresh weights. Our research provides a foundation to further study the physiological consequences of *N*. *cadamba* growth under nutrient deficiency.

## Conclusion

The present study reveals that ND inhibited the growth of *N*. *cadamba*. N and protein content in roots and leaves were both reduced. Chlorophyll and carotenoid contents were reduced as were chlorophyll fluorescence parameters. Genes encoding transcription factors, transport proteins, and oxidative stress-related proteins were significantly upregulated, suggesting that the nutrient transporting and the hormonal regulation may play an important role for *N*. *cadamba* in response to N starvation.

## Data Availability Statement

The original contributions presented in the study are publicly available. This data can be found here: RNA-seq data were submitted to (http://bigd.big.ac.cn/gsa/with) submission number: CRA004153.

## Author Contributions

A-MW and HL conceived the project. LLi, YZ, LLu, NY, YL, and MQ conducted the experiments and analyzed the data under the supervision of A-MW and HL. LLu, YZ, and MQ wrote the manuscript. All authors contributed to the article and approved the submitted version.

## Conflict of Interest

The authors declare that the research was conducted in the absence of any commercial or financial relationships that could be construed as a potential conflict of interest.

## Publisher’s Note

All claims expressed in this article are solely those of the authors and do not necessarily represent those of their affiliated organizations, or those of the publisher, the editors and the reviewers. Any product that may be evaluated in this article, or claim that may be made by its manufacturer, is not guaranteed or endorsed by the publisher.
