# Supplementary material for: Physiological and Transcriptomic Responses to Nitrogen Deficiency in Neolamarckia cadamba
Source: Front Plant Sci. 2021 Nov 23;12:747121. doi: 10.3389/fpls.2021.747121 (PMC8649893; doi:10.3389/fpls.2021.747121)
Supplement: Supplementary file 1 [file Data_Sheet_1.docx]

**Supplementary Figure 1.** **KEGG (Kyoto Encyclopedia of Genes and Genomes) and GO (Gene Ontology) enrichment analysis was performed on four sets (N6L vs C6L, N12L vs C12L, N6R vs C6R, N12R vs C12R).**

(**A**)KEGG enrichment pathways: The ordinate indicates the name of the pathway ,the abscissa indicates the rich factor, the size of the dot indicates the number of differentially expressed genes in the pathway , and the color of the dot corresponds to different q value ranges.

(**B**) The corrected p-value ≤ 0.05 was defined as prominent enriched GO terms. In biological_process, cellular_component, and molecular_function terms, biological process was the main type.

**Supplementary Figure 2. Effect of nitrogen deficiency on cell wall synthesis in *N. cadamba.***

(**A**)ND stimulates xylem secondary growth in *N. cadamba* stem.

(**B**)DEGs related to phenylalanine biosynthesis process in N. cadamba root was upregulated. A simplified biosynthetic pathway representing the most common pathways of the three major lignin units. Heatmap of columns and rows represent samples and genes, respectively. PAL: phenylalanine ammonia-lyase; C4H: cinnamate 4-hydroxylase; 4CL: 4-coumarate-CoA ligase; COMT: caffeic acid O-methyltransferase; F5H: ferulate 5-hydroxylase; CCR: cinnamoyl-CoA reductase; CCoAOMT: caffeoyl-CoA O-methyltransferase; CAD, cinnamyl alcohol dehydrogenase.

**Supplementary Figure 3. The heat map of nitrogen deficiency-induced expression of other transporter proteins(A) and transcription factors(B) is supplementary Figure 3.**

Supplementary Figure 1**
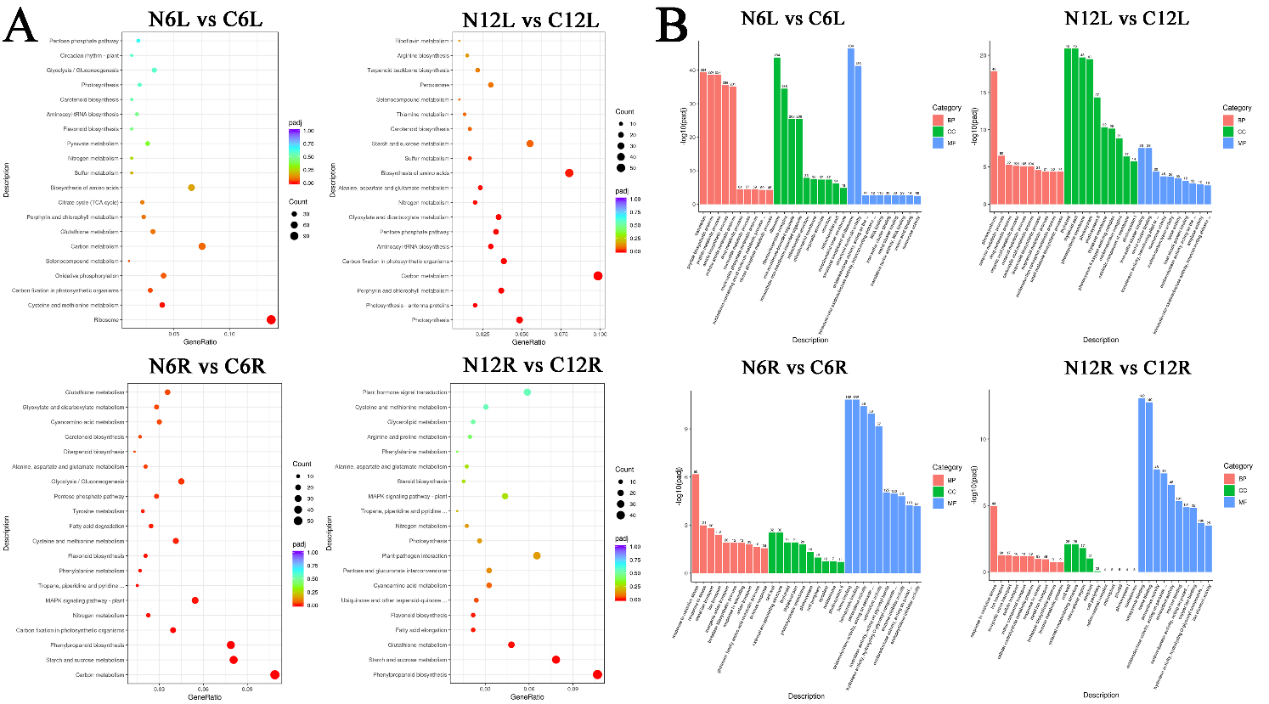
**

Supplementary Figure 2
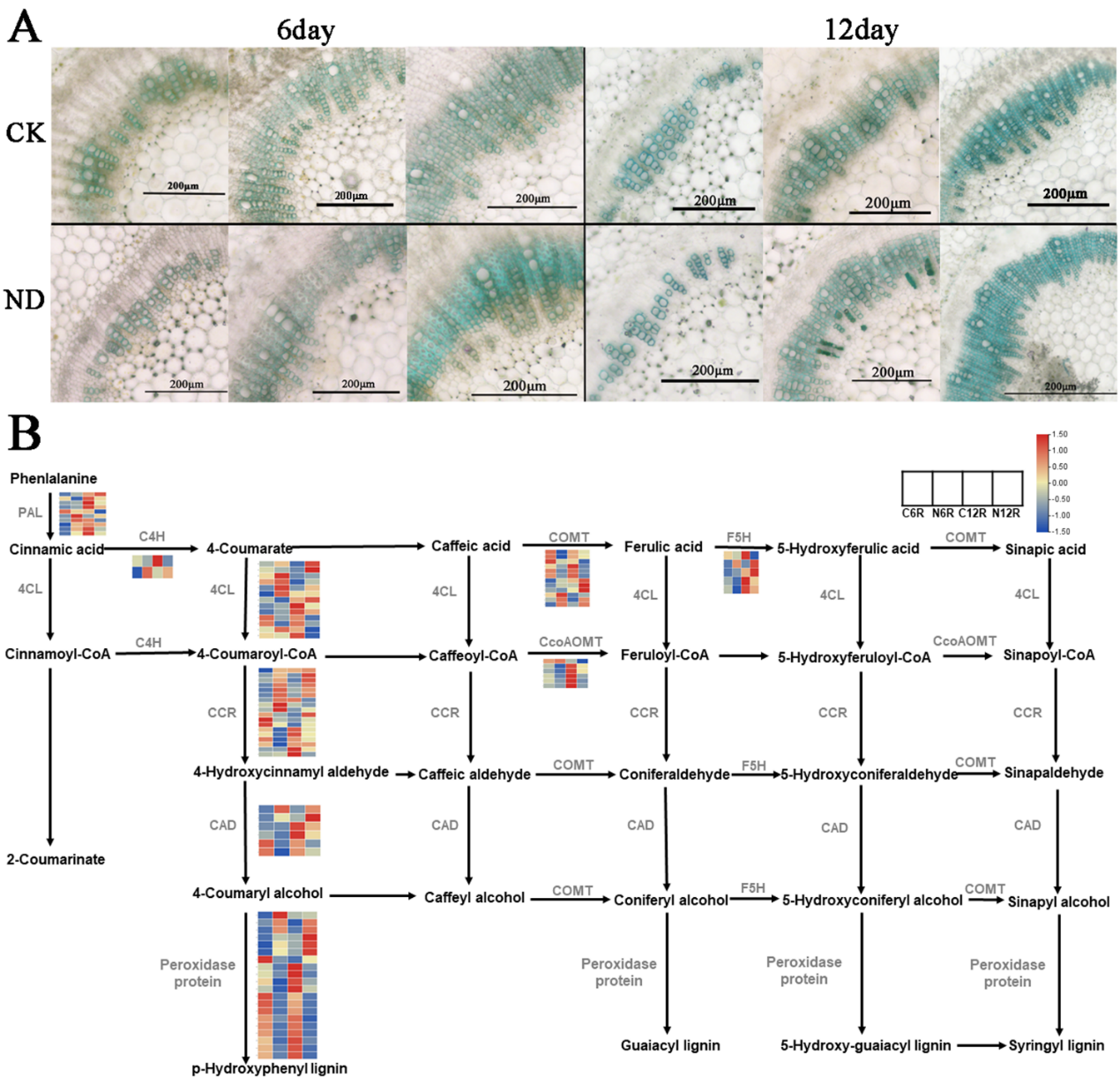
 Supplementary Figure 3**
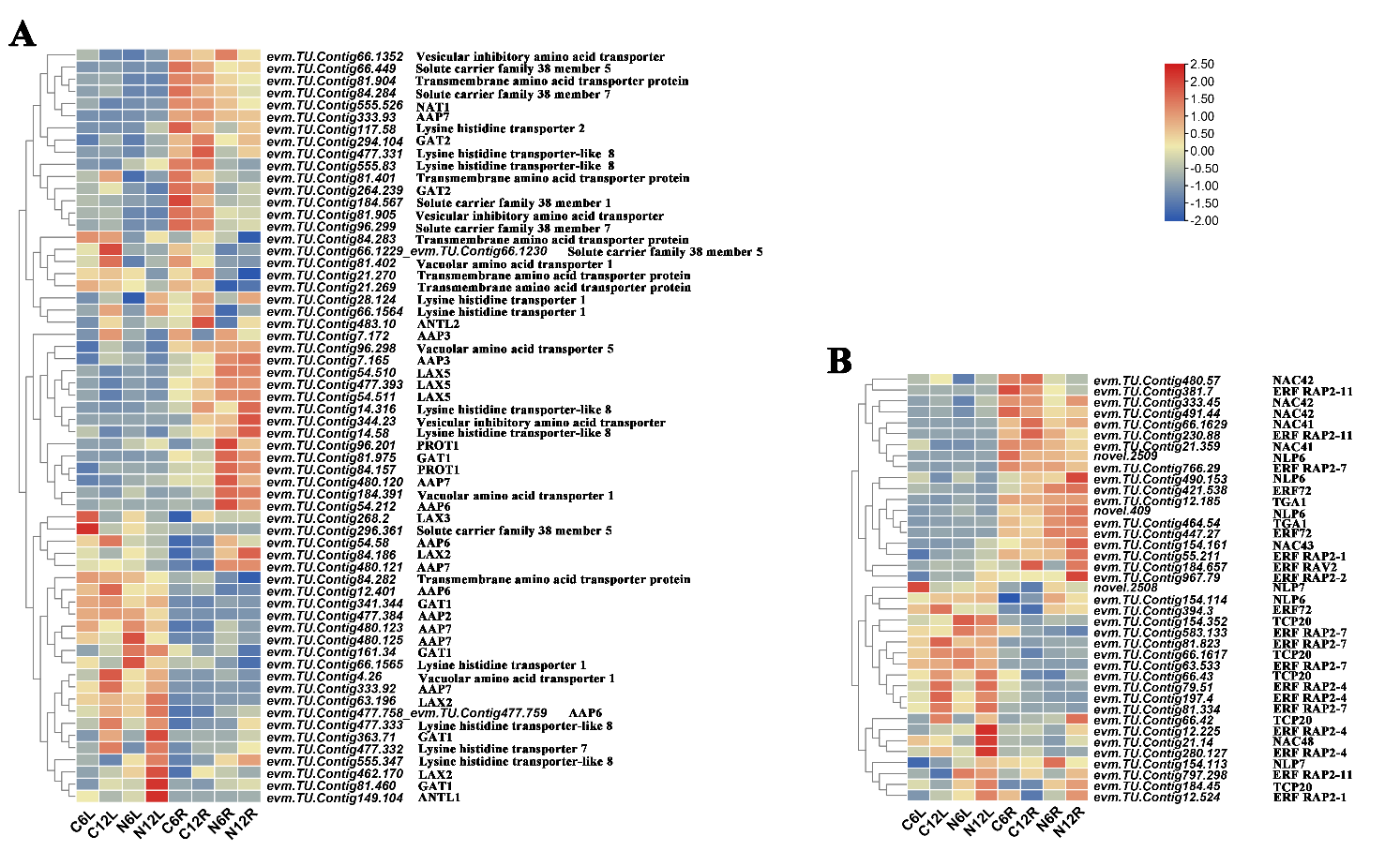
**
