# Supplementary figures and images for: Physiological and Transcriptomic Responses to Nitrogen Deficiency in Neolamarckia cadamba
Source: Front Plant Sci. 2021 Nov 23;12:747121. doi: 10.3389/fpls.2021.747121 (PMC8649893; doi:10.3389/fpls.2021.747121)

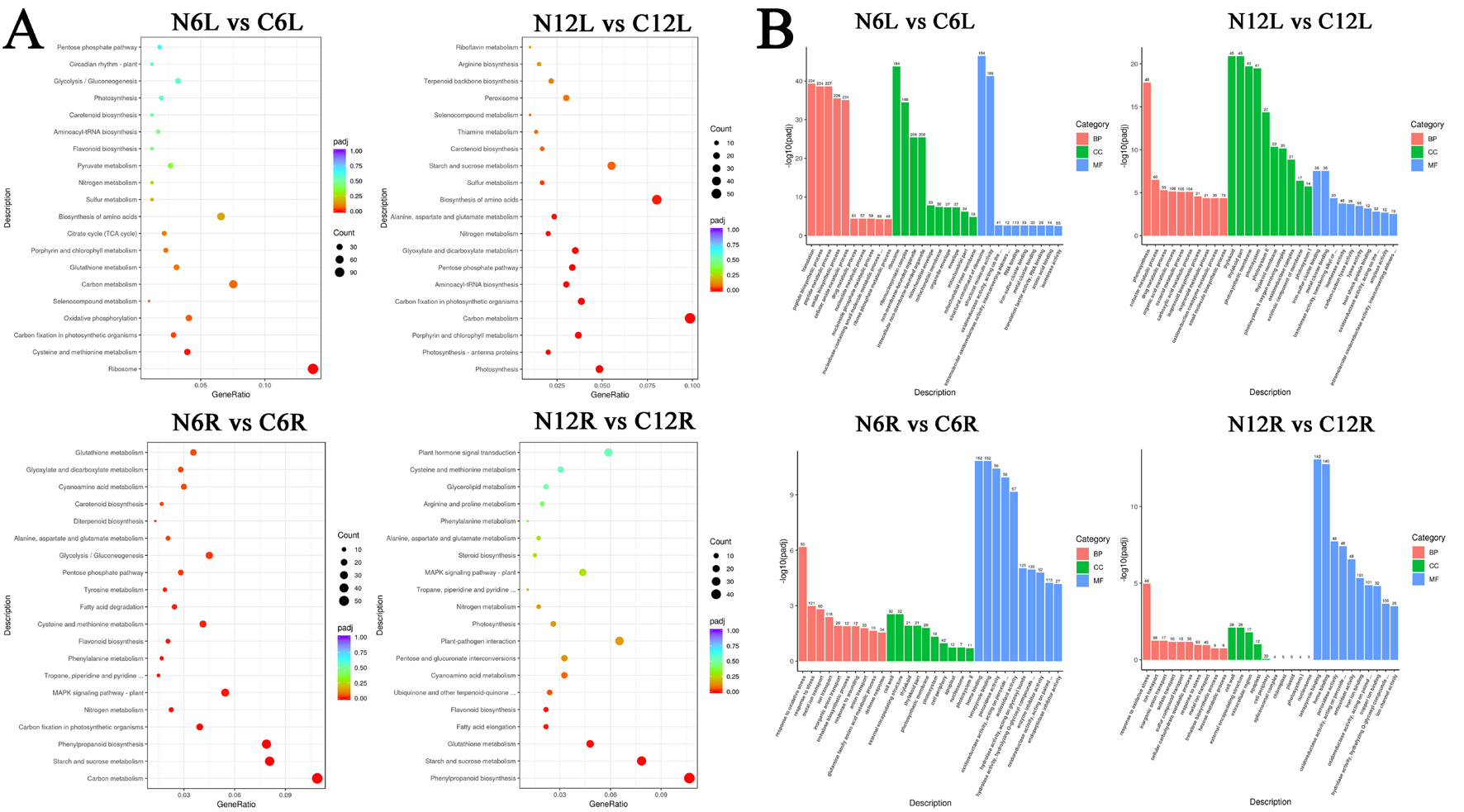

Supplement: Supplementary file 2 [file Image_1.JPEG]

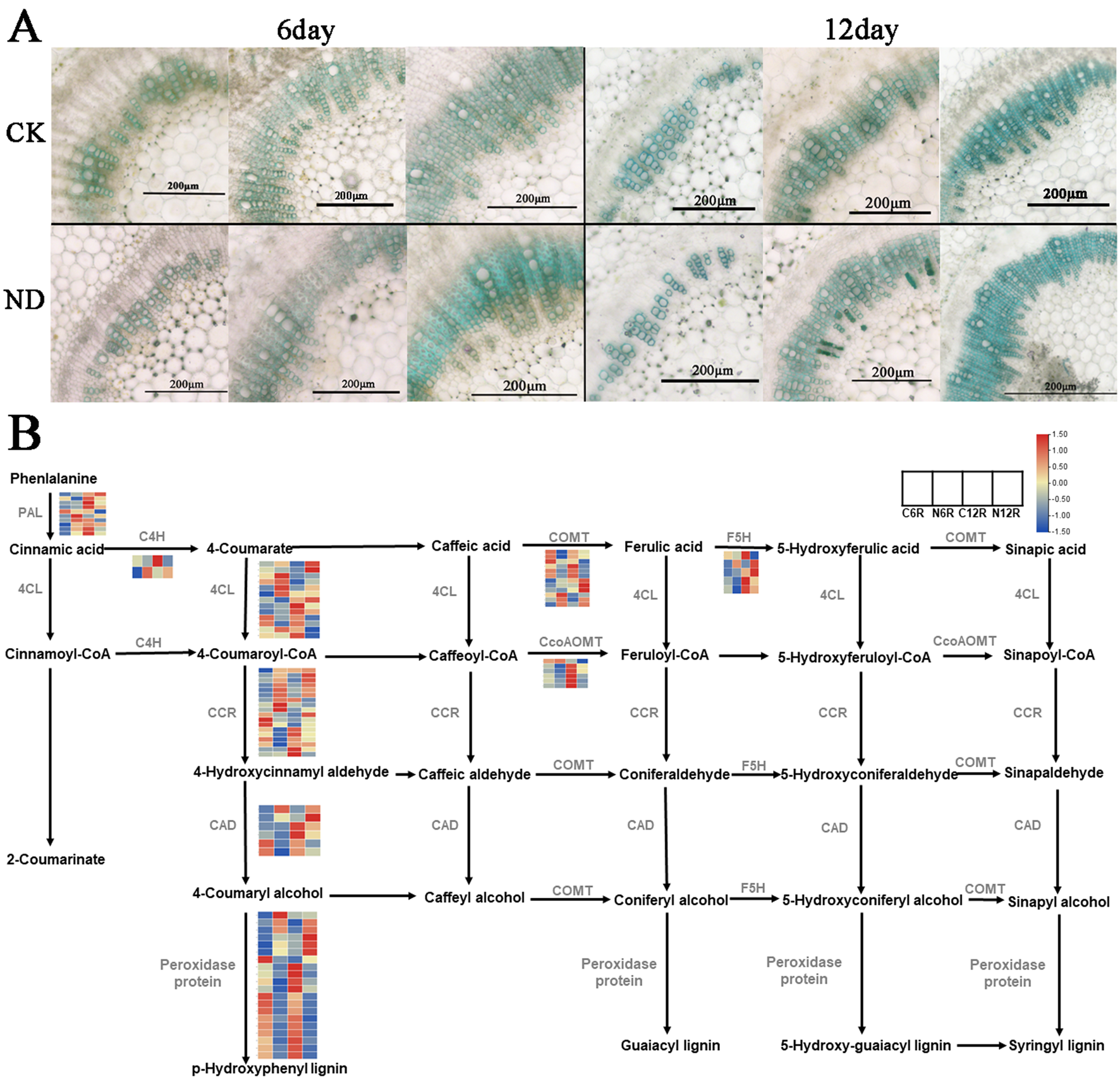

Supplement: Supplementary file 3 [file Image_2.TIF]

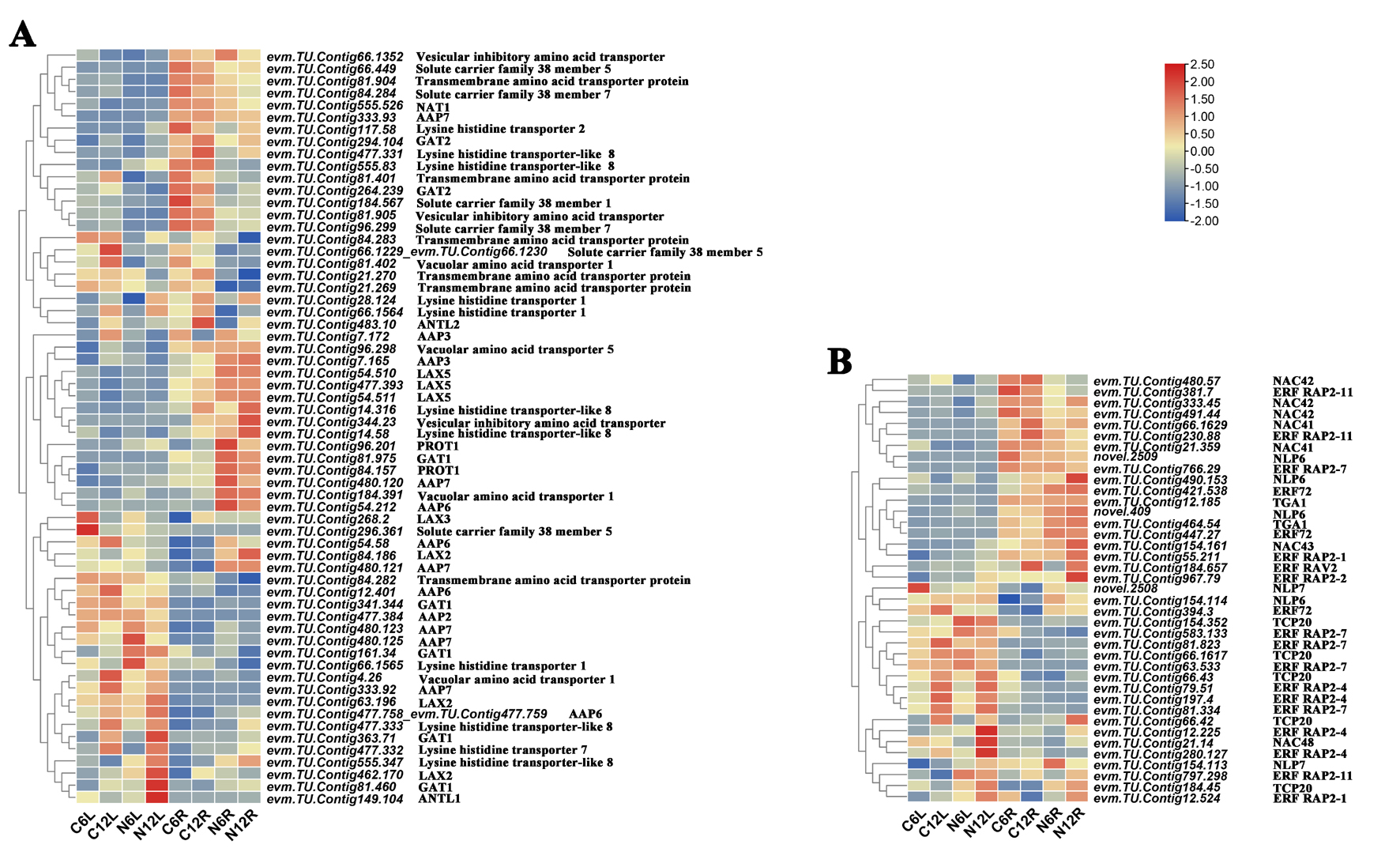

Supplement: Supplementary file 4 [file Image_3.JPEG]
