# Supplementary material for: Physiological and Transcriptomic Responses to Nitrogen Deficiency in Neolamarckia cadamba
Source: Front Plant Sci. 2021 Nov 23;12:747121. doi: 10.3389/fpls.2021.747121 (PMC8649893; doi:10.3389/fpls.2021.747121)
Supplement: Supplementary file 5 [file Table_1.docx]

| Sample_name | Raw_reads | Clean_reads | Mapped Reads | Q20(%) | GC_content(%) |
| --- | --- | --- | --- | --- | --- |
| C6L1 | 41912034 | 41303292 | 39511590(95.66%) | 98.49 | 44.27 |
| C6L2 | 44469998 | 43692622 | 41894249(95.88%) | 98.51 | 44.38 |
| C6L3 | 43358972 | 42251754 | 40490621(95.83%) | 98.5 | 44.18 |
| C6R1 | 45826534 | 44533960 | 35564797(79.86%) | 98.51 | 43.89 |
| C6R2 | 42187320 | 41336134 | 31593678(76.43%) | 98.53 | 43.68 |
| C6R3 | 41467022 | 40737042 | 33753169(82.86%) | 98.53 | 43.81 |
| C12L1 | 49926726 | 49100352 | 46982151(95.69%) | 98.64 | 44.16 |
| C12L2 | 44112942 | 43362336 | 41598478(95.93%) | 98.54 | 44.3 |
| C12L3 | 45115342 | 44270980 | 42417189(95.81%) | 98.52 | 44.27 |
| C12R1 | 42066262 | 41298388 | 36943532(89.46%) | 98.45 | 43.73 |
| C12R2 | 43891842 | 43075066 | 38497031(89.37%) | 98.12 | 43.73 |
| C12R3 | 47109044 | 46421232 | 42254886(91.02%) | 98.52 | 43.5 |
| N6L1 | 45135710 | 44465314 | 42572398(95.74%) | 98.46 | 44 |
| N6L2 | 40196976 | 39578264 | 37898356(95.76%) | 98.55 | 43.98 |
| N6L3 | 45181480 | 44136198 | 42273055(95.78%) | 98.66 | 44 |
| N6R1 | 44178710 | 42407188 | 31806034(75.0%) | 98.61 | 44.15 |
| N6R2 | 46146434 | 44940220 | 35734636(79.52%) | 98.62 | 43.98 |
| N6R3 | 45613276 | 44314598 | 33367249(75.3%) | 98.7 | 44.03 |
| N12L1 | 46657786 | 45212572 | 43351962(95.88%) | 98.7 | 44.19 |
| N12L2 | 40936232 | 40046784 | 38412747(95.92%) | 98.67 | 43.94 |
| N12L3 | 54008384 | 52572974 | 50466376(95.99%) | 98.77 | 43.89 |
| N12R1 | 45319382 | 44074948 | 33879900(76.87%) | 98.73 | 45.08 |
| N12R2 | 49115664 | 47257464 | 36343919(76.91%) | 98.74 | 45.49 |
| N12R3 | 44714392 | 43298626 | 37673773(87.01%) | 98.65 | 44.51 |

**Table S1.RNA sequencing statistics for each sample.**
