# Supplementary material for: Physiological and Transcriptomic Responses to Nitrogen Deficiency in Neolamarckia cadamba
Source: Front Plant Sci. 2021 Nov 23;12:747121. doi: 10.3389/fpls.2021.747121 (PMC8649893; doi:10.3389/fpls.2021.747121)
Supplement: Supplementary file 6 [file Table_2.docx]

|  | 6day | | | 12day | | |
| --- | --- | --- | --- | --- | --- | --- |
|  | CK | ND | TTEST | CK | ND | TTEST |
| Y(I) | 0.331±0.016 | 0.267±0.025 | 0.019* | 0.549±0.029 | 0.323±0.125 | 0.038* |
| ETR(I) | 29.733±1.361 | 23.967±2.214 | 0.018* | 49.333±2.597 | 28.967±11.243 | 0.038* |
| Y(ND) | 0.526±0.047 | 0.584±0.025 | 0.129 | 0.325±0.030 | 0.553±0.163 | 0.076 |
| Y(NA) | 0.144±0.037 | 0.149±0.036 | 0.875 | 0.125±0.018 | 0.124±0.043 | 0.972 |
| Y(II) | 0.197±0.008 | 0.169±0.019 | 0.08 | 0.288±0.044 | 0.206±0.071 | 0.162 |
| ETR(II) | 17.733±0.666 | 15.233±1.701 | 0.077 | 25.967±4.022 | 18.500±6.355 | 0.161 |
| Y(NO) | 0.255±0.008 | 0.278±0.003 | 0.009 | 0.237±0.015 | 0.246±0.035 | 0.717 |
| Y(NPQ) | 0.548±0.010 | 0.553±0.018 | 0.699 | 0.474±0.052 | 0.548±0.037 | 0.116 |
| NPQ | 2.149±0.090 | 1.993±0.068 | 0.073 | 2.009±0.314 | 2.239±0.178 | 0.331 |
| qN | 0.791±0.005 | 0.766±0.010 | 0.017* | 0.772±0.040 | 0.790±0.013 | 0.49 |
| qP | 0.396±0.020 | 0.307±0.028 | 0.011* | 0.556±0.019 | 0.383±0.114 | 0.061 |
| qL | 0.248±0.025 | 0.165±0.015 | 0.008** | 0.375±0.012 | 0.227±0.075 | 0.028* |

**Table S2. Chlorophyll fluorescence parameters.**

（* and ** denote significant differences at P < 0.05 and 0.01）
