# Supplementary material for: Physiological and Transcriptomic Responses to Nitrogen Deficiency in Neolamarckia cadamba
Source: Front Plant Sci. 2021 Nov 23;12:747121. doi: 10.3389/fpls.2021.747121 (PMC8649893; doi:10.3389/fpls.2021.747121)
Supplement: Supplementary file 7 [file Table_3.docx]

**Table S3. Primers used for qRT-PCR.**

|  | Gene ID | Annotation | Primers (5'-3') |
| --- | --- | --- | --- |
| a | *evm.TU.Contig14.55* | NRT1/ PTR FAMILY 4.5 | F: CAATGTCAGGCAGCAACCAG |
|  |  |  | R: GGCACCCAAAGATGGCAAAG |
| b | *evm.TU.Contig60.93* | High affinity nitrate transporter NRT2.5 | F: CTCACATGCGGGCATTTCAC |
|  |  |  | R: CAAGCTGTTCCCATGGCAAC |
| c | *evm.TU.Contig462.107* | Protein NRT1/ PTR FAMILY 5.6 | F: TAGTCCCGGCTTTGAAACCC |
|  |  |  | R: TCAAGTGAGGGCTTGTGTCC |
| d | *evm.TU.Contig553.93* | Transcription factor bHLH68 | F: TAGGTGGGGCGCCTAAAAAG |
|  |  |  | R: TGTCGGTCTTCCCAAAAGGG |
| e | *evm.TU.Contig967.129* | Auxin-binding protein ABP19a | F: TGCGGGTTAGTGCTGAAGAG |
|  |  |  | R: AGCATCACTCAGGACACGAC |
| f | *evm.TU.Contig66.1591* | Chloride channel protein CLC-b | F: CCTTGGTGCACATTGGAAGC |
|  |  |  | R: AAGAGTACACCGCCAACTGG |
| g | *evm.TU.Contig341.228* | Basic helix-loop-helix protein 15  BHLH15 | F: AATTCACGGTGGTGGACTCC |
|  |  |  | R: ACGTCACGGTTAGCTCACAG |
| h | *evm.TU.Contig249.46* | Basic helix-loop-helix protein 60  BHLH60 | F: ACCGCACTACTTGAGCTTCC |
|  |  |  | R: AAGCTGTGTCGGAAGGGAAG |
| i | *evm.TU.Contig345.35* | Phosphoribulokinase,PRK | F: AAGCCCTCAACTCCTCATGC |
|  |  |  | R: TGGTGGTGGTGGTTGTTCTC |
| j | *evm.TU.Contig161.34* | GABA transporter 1 | F: TTCTGGCTACTGGGCTTTCG |
|  |  |  | R: GCTCTTTGGGTCTGCAAACG |
| k | *evm.TU.Contig330.45* | Delta-aminolevulinic acid dehydratase 1, ALADH1 | F: CAAACGGCTGTTGGTCATCC |
|  |  |  | R: CCCATCGTGATGCGTTTCAC |
| l | *evm.TU.Contig63.170* | Chlorophyll synthase | F: TGTCGCAGTGAAGGGCTATG |
|  |  |  | R: TCCCGGGGCAAAGACAATAC |
| m | *evm.TU.Contig490.61* | Magnesium-chelatase subunit ChlH,CHLH | F: CAAATGCACCGGTCATTGGG |
|  |  |  | R: CTTTGGCCCCTTTTGCTTCC |
| n | *evm.TU.Contig20.155* | Gibberellin 2-beta-dioxygenase 8 | F: TGACTGCCCTTGACCCTAGA |
|  |  |  | R: AGGTGAACTTGATGCCAGGG |
| o | *evm.TU.Contig66.866* | Probable potassium transporter 17 | F: AATGATCTCCTCAGCTGCTGT |
|  |  |  | R: CTGATGCACTGCACTTGACG |
|  | *SAMDC* |  | F: GGGACCAACTGGATGAAA |
|  |  |  | R: TGTAAGGGAAGACAAAGAG |
